# Zinc as a Therapeutic Agent in Bone Regeneration

**DOI:** 10.3390/ma13102211

**Published:** 2020-05-12

**Authors:** J. Patrick O’Connor, Deboleena Kanjilal, Marc Teitelbaum, Sheldon S. Lin, Jessica A. Cottrell

**Affiliations:** 1 Department of Orthopaedics, Rutgers-New Jersey Medical School, Newark, NJ 07103, USA; linss@njms.rutgers.edu; 2 School of Graduate Studies, Rutgers, the State University of New Jersey, 185 South Orange Avenue, Newark, NJ 07103, USA; dk731@gsbs.rutgers.edu (D.K.); mht45@gsbs.rutgers.edu (M.T.); 3 Department of Biological Sciences, Seton Hall University, 400 South Orange Avenue, South Orange, NJ 07079, USA; jessica.cottrell@shu.edu

**Keywords:** zinc, bone regeneration, bone healing, osteogenesis, therapeutics, bone biology, osteoclasts, osteoblasts, chondrocytes

## Abstract

Zinc is an essential mineral that is required for normal skeletal growth and bone homeostasis. Furthermore, zinc appears to be able to promote bone regeneration. However, the cellular and molecular pathways through which zinc promotes bone growth, homeostasis, and regeneration are poorly understood. Zinc can positively affect chondrocyte and osteoblast functions, while inhibiting osteoclast activity, consistent with a beneficial role for zinc in bone homeostasis and regeneration. Based on the effects of zinc on skeletal cell populations and the role of zinc in skeletal growth, therapeutic approaches using zinc to improve bone regeneration are being developed. This review focuses on the role of zinc in bone growth, homeostasis, and regeneration while providing an overview of the existing studies that use zinc as a bone regeneration therapeutic.

## 1. Zinc Biodistribution and Main Intracellular Roles

Zinc is an essential mineral required for several cellular processes in the body. In a typical 70 kg human, zinc is the sixth most abundant metal (2.3 g) behind calcium (1 kg), potassium (140 g), sodium (100 g), magnesium (19 g), and iron (4.2 g) and is more abundant than rubidium (0.68 g), strontium (0.32 g), and other trace metals [1]. Zinc is normally obtained from the diet and the recommended daily consumption is 15 mg for adults [2]. Serum zinc levels in humans are about 15 µM or 100 µg/dL (75–125 µg/dL) [3]. In the blood, zinc is bound by albumin and α_2_-macroglobulin, which reduces the amount of free zinc in the serum to less than 1 nM [4,5,6,7,8]. Acute zinc toxicity requires substantial systemic exposure and has been measured in rats at an oral LD_50_ of 237–623 mg/kg and an intraperitoneal LD_50_ of 28–73 mg/kg [9].

Zinc transporter ZIP4 and ion channel TRPM7 appear to be critical for zinc absorption in the intestine. Mutations in ZIP4 (*Slc39a4*) are associated with acrodermatitis enteropathica, a genetic disorder that manifests at weaning and is lethal unless treated by dietary zinc supplementation, indicating that ZIP4 is necessary for intestinal zinc absorption [10,11,12,13,14,15]. Conditional ablation of ZIP4 in mouse intestine produced an acrodermatitis enteropathica phenotype that could be treated with dietary zinc [16]. Targeted deletion of *Trpm7* in the intestine of mice causes post-natal lethality by day 10 with decreased levels of zinc and calcium in serum and bone [17]. Loss of ZIP4 in the intestine may initially affect Paneth cells leading to some indirect effects on zinc absorption by the intestine [16].

At the molecular level, approximately 10% of the mammalian proteome binds zinc [18]. Many enzymes use zinc as a co-factor while other proteins use zinc to stabilize structural conformations. For instance, zinc binding to protein Cys_2_His_2_ motifs stabilizes characteristic zinc-finger structures that interact with DNA [19]. In other cases, zinc can differentially bind to a protein to direct the function of that protein. For instance, metal response element-binding transcription factor 1 (MTF-1) responds to increased intracellular zinc levels by localizing in the nucleus where this transcription factor binds to target genes to affect transcription [20,21,22,23,24]. Zinc binding appears to inhibit a nuclear export signal in MTF-1 and thereby enables prolonged nuclear residency of MTF-1 when intra-nuclear zinc concentrations are sufficiently high [25]. MTF-1 is an evolutionarily conserved protein and loss of MTF-1 causes embryonic lethality in mice [26]. 

Intracellular zinc levels are tightly controlled. Though zinc can be transported through certain calcium channels [27], cells also have a large repertoire of zinc transporters and binding proteins to regulate intracellular zinc [28,29]. Specifically, ZIP1 through ZIP14 (*Slc39a1*–*Slc39a14*) are zinc transporters that move zinc into the cytosol from extracellular or intracellular organelle zinc stores. In contrast, ZnT1-ZnT10 (*Slc30a1*–*Slc30a10*) are zinc transporters that move zinc from the cytosol into intracellular organelles or into the extracellular space. Targeted null mutations in ZIP13, ZIP14, and ZnT5 are associated with skeletal defects in mice [30]. Within the cytosol, zinc is also bound by metallothioneins (MT1–MT4) to buffer and regulate cytosolic zinc levels [31]. 

Zinc can affect many signaling pathways including insulin and IGF-1 [32,33,34,35,36]; PKA, PKC and MAPK [35,37,38,39]; NF-kB [40,41,42], NFAT1c [41]; and JAK-STAT [42]. Zinc appears to promote insulin-related and IGF-1 related signaling by affecting protein tyrosine phosphatase activity [43,44,45]. However, there does not appear to be a canonical zinc-signaling pathway. For instance, Grp39 is a G-protein coupled receptor that responds to extracellular zinc levels to regulate pancreatic β-cell insulin secretion and promotes gastro-intestinal function and integrity [46,47,48]. However, mice that are homozygous for a targeted mutation in Grp39 are viable with an age-related deficit in insulin secretion [49,50]. Whether Grp39 responds to zinc levels in the skeletal cells or in bone has not been determined.

Though much is known about the biology of zinc, how zinc affects skeletal growth and regeneration is still poorly understood. The following summarizes our current understanding of zinc effects on different bone cell types and experiments showing that zinc can be used to promote bone formation and regeneration.

## 2. Role of Zinc in Skeletal Growth

**Figure 1:**
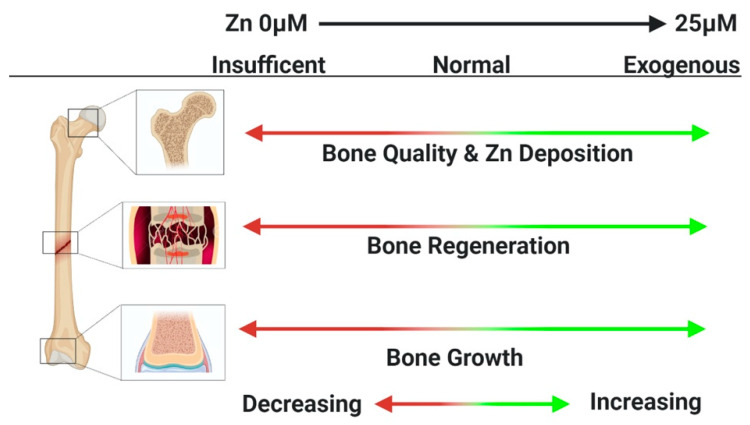
Roles of Zinc in the Skeleton.

Dietary zinc deficiency has been linked to impaired skeletal development and bone growth in humans and animals (Figure 1) [51,52,53,54,55,56,57,58,59,60,61,62]. Early experiments with rats to determine if dietary zinc was essential for growth were complicated by co-reduction of essential vitamins and amino acids in zinc-depleted media. Still, these experiments found reduced growth in juvenile rats fed a zinc-deficient diet when compared to control cohorts; this zinc deficiency could be restored by addition of zinc into the diet [63,64]. In 1961, Prasad et al. described 11 men (age 14–21) with short stature, hypogonadism, iron deficient anemia, hepatosplenomegaly, and a long history of geophagia (eating dirt) that was reversed by oral iron therapy and a well-balanced diet [51]. Prasad et al. speculated that the dwarfism and hypogonadism observed in these patients were caused by zinc deficiency. In a follow-up study of 40 patients with dwarfism and hypogonadism, Sandstead et al. confirmed that these patients were zinc-deficient and that dietary zinc therapy improved growth to a greater extent than iron therapy [65]. Consistent with reduced skeletal growth, circulating levels of growth hormone and IGF-1 are reduced during zinc deficiency [33,66].

Zinc is located at sites of tissue calcification, including osteons and calcified cartilage [67]. Bone tissue zinc levels increase as bone mineralization increases [28,68]. Alkaline phosphatase uses zinc as a co-factor and is involved in bone mineralization [69,70,71,72]. In vivo, turkey poults fed a zinc-deficient diet show reduced growth, shortened long bones, and reduced alkaline phosphatase activity in proportion to dietary zinc levels [73]. In vitro, zinc concentrations from 7–20 nM stimulate alkaline phosphatase activity but at zinc concentration above 5 µM, zinc can inhibit alkaline phosphatase activity [72,74,75]. These observations support a direct role for zinc in extracellular matrix mineralization via alkaline phosphatase and suggest that impaired osteoid mineralization or calcified cartilage formation associated with endochondral ossification may contribute to impaired bone growth during zinc deficiency.

## 3. Zinc and Bone Homeostasis

Typically, zinc distribution in the body is about 57% in muscle, 29% in bone, 6% in skin, 5% in liver, 1.5% in the brain, and 0.1% in blood plasma [76]. Zinc homeostasis is primarily controlled in the gut by inversely altering the amount of endogenous zinc excreted relative to the exogenous amount of zinc absorbed [77,78,79]. The gut maintains body zinc levels at normal homeostatic levels over a wide range of dietary zinc levels [78]. In contrast to the gut, excretion of zinc from the kidney remains relatively constant despite changes in dietary zinc amounts [78]. Only when human dietary zinc levels drop below about 3 mg/day is excretion from the kidneys reduced [79,80].

Lack of dietary zinc can alter the typical distribution of zinc stored in the body. Zinc preferentially localizes in bone (Figure 1) [81]. So, when dietary zinc is limited, serum zinc levels appear to be maintained at a vital level by preferential mobilization of zinc from bone [82,83,84,85]. The physiological mechanism by which low serum zinc triggers release of zinc from bone is unknown. Murray and Messer altered dietary intake of calcium and zinc in rats that had been treated with radio-labelled calcium, zinc, and tetracycline to assess whether low dietary zinc would trigger bone resorption [86]. While low dietary calcium stimulated bone resorption, low dietary zinc, at least at the levels used in this study, failed to stimulate bone resorption. Interestingly, the zinc released from bone was quickly re-absorbed by the bone independently of mineral deposition. In a few studies, reduced serum zinc levels have been associated with post-menopausal osteoporosis [87,88]. However, studies comparing age-matched osteoporotic and non-osteoporotic women found elevated or similar serum zinc levels between cohorts, though the osteoporotic women consistently had increased urinary excretion of zinc [89,90]. Thus, whether and how low serum zinc levels may trigger release of zinc from bone needs to be further investigated.

Serum zinc levels do respond to physiological signals. Zinc is known to stimulate insulin secretion from pancreatic β cells, modulate insulin action, and affect glucose metabolism [91,92]. Consequently, stimulation of insulin secretion by consumption of a glucose bolus, temporarily reduces serum zinc [3]. Physiological stressors including surgical trauma, hemorrhagic shock, thermal injury, and bone fracture all can cause temporary reductions in serum zinc levels [93,94,95,96,97]. Interestingly, while serum zinc levels decrease following a traumatic injury, zinc levels rise at the site of injury [98,99,100,101,102]. Zinc can also modulate innate or adaptive immune responses [103,104]. Whether the temporary reduction in serum zinc in response to physiological stressors is a means to modulate the immune response requires further investigation.

In addition to the role of zinc in normal body and skeletal growth, dietary zinc is also important for bone quality (Figure 1). When juvenile male Sprague-Dawley rats were fed a zinc-deficient diet for 42 days, the rats had lower body weight and developed osteopenia characterized by a 45% reduction in cancellous bone, a 30% reduction in osteoblast surface area and a 38% reduction in osteoclasts [56]. Conversely, when streptozotocin-induced Type 1 diabetic rats were treated with daily oral doses of zinc sulfate (250 μg/kg/day) for 8 weeks, femoral bone volume, trabecular thickness, and bone mineral density were significantly improved even though the rats remained hyperglycemic [105].

## 4. Zinc Effects on Osteoblasts

**Figure 2:**
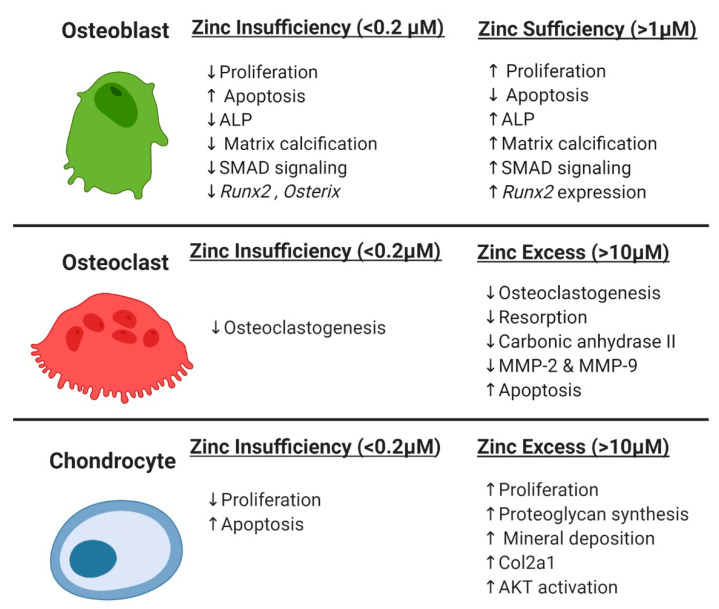
Zinc Effects on Skeletal Cells.

Given the necessity for zinc in skeletal development and growth, considerable research has been performed assessing the effects of zinc on osteoblast activity (Figure 2). A common finding was that zinc promotes osteoblast proliferation in established and primary osteoblast cell models [32,37,106,107,108,109,110,111,112] and in animals [54]. A second common finding was that zinc increased alkaline phosphatase (ALP) activity in vitro [32,37,106,109,110,111,112,113,114], in organ culture [113], and in vivo [53,54,115,116]. A third common finding was that the effects of zinc on osteoblast proliferation, ALP activity, and other osteogenic processes showed a bi-phasic dose response. The positive effects of zinc on osteoblast activity occur over a narrow dose range (1–50 µM); doses above that range inhibit osteogenic activity, and below that range have no measurable effects [37,108,112,113,117,118,119,120]. Only very high doses of zinc (600 and 900 µM) were found to be cytotoxic [121]. 

Several studies found that addition of zinc to tissue culture media or by growing cells on a substratum containing zinc increases *Runx2* expression [114,122,123,124,125,126]. Similarly, *Runx2* expression was reduced in bones from rats fed a zinc-deficient diet [54]. As *Runx2* expression is essential for osteoblast differentiation [127,128], the results indicate that zinc can be osteo-inductive. The mechanism by which zinc induces *Runx2* expression is less clear. Cho and Kwun found that when MC3T3-E1 (subclone 4) cells were cultured in 1 µM zinc, SMAD-1 activation and BMP-2 expression were reduced along with *Runx2*, Osterix, and other osteogenesis related genes [125]. However, when zinc increased to 15 µM, BMP-2 expression, SMAD-1 activation, and *Runx2* expression increased, suggesting that zinc induces *Runx2* through canonical BMP-2 signaling. In contrast, Park et al. noted that zinc induced *Runx2* expression was reduced by inhibition of PKA with H-89, suggesting that zinc induced *Runx2* expression through a cAMP mediated process [126].

Effects of zinc treatment on matrix deposition and calcification by osteoblasts were more complex. In vivo, matrix-related gene expression was reduced in the bone of rats fed a zinc-free diet [54]. In vitro, Kwun et al. and Alcantra et al. showed that culturing MC3T3-E1 cells in zinc-deficient media reduced ALP activity, expression of matrix-related genes (such as *Col1a1*, osteopontin, and osteocalcin), and mineralized matrix deposition [111,114]. Cerovic et al. noted that 10 µM zinc increased ALP activity and mineralized nodule formation by SaOS2 cells but that 50 µM zinc inhibited ALP activity and mineralization [118]. Seo et al. observed increased collagen synthesis with increased media zinc levels (up to 25 µM) in MC3T3-E1 cultures [110]. Togari et al. noted that treating MC3T3-E1 cultures with 10 µM zinc increased ALP activity without enhancing calcium deposition while higher zinc doses (50 and 100 µM) inhibited calcium deposition [113]. In contrast, Yamaguchi and colleagues found that treating MC3T3-E1 cells with zinc had no effect on matrix gene expression [122,129]. The differential effects of zinc on matrix deposition may reflect differences in the cell culture models or zinc salts used. 

Zinc also protects osteoblasts from apoptosis. When MC3T3-E1 cells were cultured in zinc-deficient or zinc-free media, apoptosis rates increased from 7% in normal media, to 75 and 90% respectively [130]. Apoptosis was associated with increased cytoplasmic cytochrome C levels indicative of a mitochondrial activated apoptosis [35,130,131]. The addition of zinc to culture media protected against H_2_O_2_-induced apoptosis by increasing ZnT7 (*Slc30a7*) expression which lead to activation of extracellular-signal-regulated kinase (ERK) and protein kinase B (AKT) [35,38]. ZnT7 transports cytoplasmic zinc into the Golgi apparatus [132]. In addition to the above effects, zinc has been noted to promote osteoblast cell spreading [120], attachment [109], and chemotaxis [133]. 

## 5. Zinc Effects on Osteoclasts

As an essential trace metal, zinc is necessary for osteoclastogenesis (Figure 2). When 5 week old female Wistar rats were fed a zinc-free diet for 3 weeks and compared to controls fed a normal diet (either ad libitum or pair-fed), 33% less zinc was found in the serum and 25% less zinc was found in the bone of the zinc-starved rats [54]. The number of osteoclasts observed in the distal femur growth plate of the zinc-starved rats was 50% less than in the control rats. Bone extracts from the zinc-starved rats had reduced tartrate-resistant acid phosphatase (TRAP) and cathepsin K activity. However, the reduction in osteoclasts was accompanied by a decrease in osteoblasts suggesting that decrease in osteoclastogenesis observed in the zinc-starved rats occurred indirectly through osteoblast-osteoclast coupling, though further investigation is needed.

In contrast, in vitro models of osteoclastogenesis using mouse RAW264.7 cells or bone marrow cells from mice or rats found that exogenous zinc inhibits osteoclastogenesis in a dose dependent manner [41,134,135,136,137,138,139,140,141]. Dose response effects varied with some reports suggesting that sub-nanomolar concentrations of zinc could inhibit osteoclastogenesis [142], whereas others found that inhibition of osteoclastogenesis only occurred at concentrations greater than 1 µM [139,140]. Osteoclastogenesis was also inhibited when bone marrow cells were cultured directly on zinc-containing ß-tricalcium phosphate or hydroxyapatite scaffolds [143,144].

Zinc inhibition of osteoclastogenesis appears to occur through direct and indirect mechanisms. NFATc1 activity is critical for osteoclastogenesis [145,146,147,148]. Park et al. found using bone marrow and RAW264.7 models of osteoclastogenesis that zinc inhibited calcineurin phosphatase activity, which reduced nuclear levels of dephosphorylated NFATc1 and impaired osteoclastogenesis [41]. Yamaguchi and Weitzmann suggested that zinc inhibits osteoclastogenesis directly by inhibiting NF-κB signaling [141]. Yamaguchi and Weitzman found that 10, 100, and 250 µM ZnSO_4_ treatment inhibited receptor activator of nuclear factor κB ligand (RANKL)-induced osteoclast differentiation of RAW264.7 cells and that the 100 and 250 µM ZnSO_4_ treatments also inhibited RANKL-induction of an NF-κB-luciferase reporter. Similarly, Hie and Tsukamoto noted that expression of the receptor activator of NF-κB (RANK) declined sharply in rats administered additional zinc [140]. As activation of the NF-κB pathway can promote osteoclastogenesis [149], the results indicate another potential mechanism by which zinc could impair osteoclast differentiation. Fong et al. found that increased dietary zinc was associated with decreased RANKL expression suggesting zinc can also indirectly inhibit osteoclastogenesis by reducing RANKL expression in other cell types [150]. Thus, zinc appears to impair osteoclastogenesis through direct and indirect mechanisms.

ZIP14 (*Slc39a14*) imports zinc into cells and consistent with a negative effect of zinc on osteoclasts, Sasaki et al. found that mice homozygous for a targeted mutation in ZIP14 have normal osteoclastogenesis but with increased osteoclast activity [151,152]. In contrast, no effect on osteoclasts was detected in mice lacking the zinc receptor (*Grp39*) [153]. 

Zinc has been demonstrated to modulate osteoclast activity. Reduced TRAP activity was associated with culturing osteoclasts on zinc-substituted ß-tricalcium phosphate discs or when administering rats additional zinc in their drinking water [140,143]. Reduced osteoclast resorption activity was associated with culturing osteoclasts in 100 µM zinc [41,154]. Interestingly, Hadley et al. observed increased TRAP activity in rats fed increasing amounts of zinc (2.5 to 30 mg zinc per kg of chow), but decreased carbonic anhydrase II, MMP-2, and MMP-9 activity [53]. Similar negative effects on osteoclast activity were observed when rabbit osteoclasts were cultured on zinc-substituted tricalcium phosphate discs [155]. As TRAP is expressed early during osteoclasts differentiation and maturation, Hadley et al. speculated that zinc may impair osteoclast maturation [53].

Zinc also can induce osteoclast apoptosis. Using purified rabbit osteoclasts cultured on tricalcium phosphate discs containing 0.3, 1.3, and 6.8 ppm zinc (ZnTCP), Li et al, found that osteoclast apoptosis increased from about 5% on the 0.3 ppm ZnTCP to over 30% on 6.8 ppm ZnTCP after 24 h in culture [156]. In contrast, β-tricalcium phosphate (TCP) discs containing similar levels of magnesium had no effect on osteoclast apoptosis. Yamada et al. also observed increased rabbit osteoclast apoptosis associated with culturing the osteoclasts on zinc-substituted tricalcium phosphate discs [155]. Apoptosis was associated with disruption of actin ring formation in both studies. 

While some level of zinc is necessary for osteoclastogenesis, the available studies indicate that excessive zinc inhibits osteoclastogenesis, impairs osteoclast resorptive activity, and promotes osteoclast apoptosis. Given the positive effects of zinc on osteoblast activity as described above and the negative effects of zinc on osteoclast activity, zinc may have a role in treating or preventing osteopenia or osteoporosis [87,157,158,159,160].

## 6. Zinc Effects on Chondrocytes

Chondrogenesis is essential for fetal bone development and normal endochondral osteogenesis during bone growth [161,162,163]. Consistent with impaired bone formation and reduced growth in zinc-deficient animals and humans [51,57,61,65], zinc also affects chondrogenesis (Figure 2). Growth of chick embryo long bone cartilage anlage positively correlates with the amount of zinc in organ culture media [62]. In contrast, growth plate chondrocyte proliferation is reduced and apoptosis is increased in newly hatched chicks fed a zinc-deficient diet (10 mg Zn/kg chow) as compared to pair-fed, newly hatched chicks fed a normal diet (68 mg Zn/kg) [164]. In vitro, addition of zinc to culture media increases primary chondrocyte proliferation from chickens and rabbits [165,166]. The effects of zinc on chondrocyte activity was bi-phasic such that high levels of zinc either produced no positive effect or were cytotoxic [165]. The mechanisms through which zinc affects chondrocytes and cartilage are not well defined. Both Burgess et al. and Huang et al. found that zinc can increase AKT activation in chondrocytes, which could account for the positive effects of zinc on proliferation [167,168].

In addition to the effects on chondrocyte proliferation, zinc was found to affect chondrocyte matrix synthesis in several studies. Using rabbit chondrocytes, Kirkpatrick et al. found that 50 µM zinc promoted chondrocyte proliferation but inhibited proteoglycan synthesis. Pasqualicchio et al. similarly found that while 10 µM zinc had no effect, 100 µM zinc inhibited proteoglycan synthesis by porcine chondrocytes [169]. Lichtfield et al. and Kirsch et al. found that Zn concentrations above 25 µM inhibited matrix mineralization in chick growth plate chondrocytes [170,171]. Koyana et al. found that 1–50 µM Zn increased collagen synthesis in primary bovine chondrocytes, but had no effect on proteoglycan synthesis [172]. More recently, Burgess et al. found that 100 µM Zn increased *Col2a1* expression as well as proteoglycan and mineral deposition in the mouse teratocarcinoma derived ATDC5 chondrocytic cell line [167]. The variation in responses to Zn and the Zn doses used likely reflects the source of the chondrocytes (growth plate vs. articular cartilage), developmental stage (fetal, newborn, or adult), as well as culture methods.

Variation in zinc levels have also been associated with pathological changes in cartilage and bone. Ovesen et al. measured serum, urine, and bone levels of zinc in 20 women undergoing hip arthroplasty for osteoarthritis and another 20 women undergoing hip arthroplasty for osteoporosis-related hip fractures [87]. Remarkably, serum levels of zinc were significantly elevated in the osteoarthritis cohort as compared to the osteoporosis cohort while urine levels of zinc were significantly reduced in the osteoarthritis cohort as compared to the osteoporosis cohort. In contrast, bone zinc levels were nearly identically between the cohorts. Interestingly, Huang et al. found that orally dosing rats with 1.6 or 8 mg/kg of zinc significantly reduced knee joint articular cartilage damage following monosodium iodoacetate injection to induce osteoarthritis [168]. 

Increased systemic zinc levels may affect cartilage damage indirectly by altering the inflammation response [40,173,174,175,176]. More recently, Kim et al. illustrated a more direct role for zinc in the pathogenesis of osteoarthritis [177]. Kim et al. found that intracellular zinc levels and ZIP8 expression were elevated in human chondrocytes obtained from osteoarthritic tissue. In follow-up experiments, Kim et al. found that overexpressing ZIP8 in chondrocytes increased expression of multiple catabolic enzymes capable of promoting cartilage destruction, including MMP3, MMP9, MMP12, MMP13, and ADAMTS5. The increased expression of these catabolic enzymes was correlated with increased nuclear localization of metal regulatory transcription factor-1 (MTF-1). Conversely, conditional knockout of ZIP8 in chondrocytes reduced cartilage destruction in mice following destabilization of the medial meniscus (a standard model for inducing osteoarthritis) while lowering intracellular zinc levels and reducing expression of MMP3, MMP13, and ADAMTS5 in the articular chondrocytes. Thus, zinc may have direct and indirect roles in osteoarthritis and other chronic inflammatory disorders. 

## 7. Using Zinc to Promote Bone Regeneration

Methods are needed to improve bone regeneration following traumatic injuries, arthrodesis, or arthroplasty. Reduced healing time would enable patients to return to their normal activities sooner, potentially reducing physical therapy associated with muscle disuse, and reducing complications caused by temporary immobility. Enhancing bone regeneration would reduce rates of delayed healing, non-unions, and failed fusions that affect a significant proportion of all patients and especially those with comorbidities, including advanced age and diabetes. 

**Figure 3:**
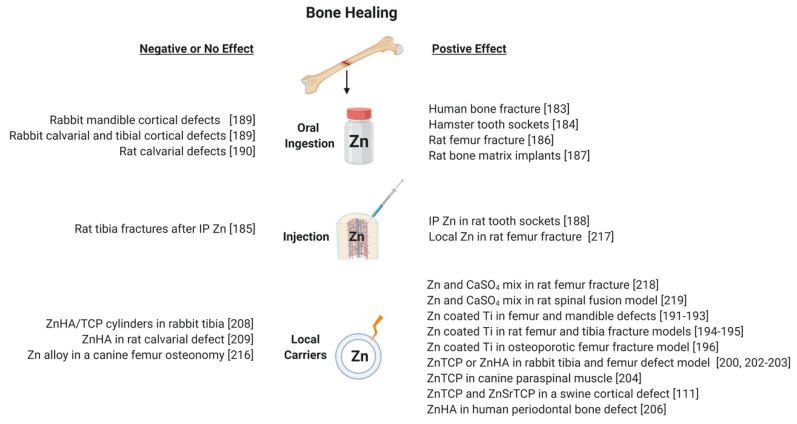
Promoting Bone Regeneration with Zinc.

Despite the known effects of zinc on skeletal cells and bone homeostasis, the potential use of zinc to promote bone regeneration has received proportionally less research attention. However, from the available results, zinc appears to promote bone formation and bone regeneration but within a limited dose range, such that too little or too much zinc is ineffective (Figure 1 and Figure 3). Conversely, when zinc dosing is optimal, the positive effects on bone regeneration appear to be sufficient to positively impact clinical outcomes.

One therapeutic approach would be to increase zinc at sites of bone regeneration by oral ingestion or other systemic delivery of a zinc compound (Figure 3). The oral and systemic tolerance for zinc is high [2,178], suggesting systemic zinc treatment may be a viable therapeutic approach. Furthermore, multiple studies discussed above have documented the accumulation of zinc in bone tissue and specifically at sites of mineralizing tissue [98,179,180]. Thus, there is a natural predilection for zinc to localize at sites of bone formation. 

By treating fracture patients per os with 50 mg/day of zinc as zinc sulfate, Sadhigi et al. reported that 24 of 30 fracture patients showed radiological evidence of fracture healing after 60 days as compared to 15 of 30 fracture patients treated with placebo [181]. In pre-clinical studies, McCray et al. reported accelerated healing in tooth sockets of Syrian hamsters (70–80 g) treated with 1 mg zinc sulfate per day in their diet [182]. Battisone et al. also found that systemic zinc administration (250 µg Zn per day by intraperitoneal injection) accelerated healing of tooth sockets in rats [183]. Similarly, oral administration of 100 mg/kg of zinc acexamate improved multiple biochemical indices of fracture healing in a Wistar rat femur fracture model [184]. Belanger et al. also reported that rats fed a zinc-supplemented diet showed improved ectopic bone formation induced with allogenic bone matrix as compared to rats fed a zinc-deficient diet [185]. Conversely, daily intraperitoneal injections of 0.6 mg of zinc-aspartate (119 μg zinc) in rats failed to improve healing of tibia fractures [186].

In contrast, studies using animal models of intramembranous ossification failed to find a significant effect of dietary zinc supplementation on improved bone healing. Abrisham et al. examined healing of shallow (2 mm deep) calvaria and tibia cortical defects as well as unicortical and bicortical defects in the mandible of rabbits treated with 100 mg zinc sulfate per day [187]. After 2 months of healing, no significant difference was detected between the control and zinc treated groups. Similarly, Jones et al. found that supplementing the diet of rats with zinc failed to significantly enhance healing of calvarial defects filled with bone graft [188]. 

Another therapeutic approach would be to deliver zinc locally to sites in need of bone formation (Figure 3). As detailed below, this approach has been successfully applied in pre-clinical and clinical models using zinc alloys, zinc-coated materials, zinc-substituted calcium salts, and direct application of zinc salts with and without carriers. 

Bone formation is enhanced around materials with altered surface compositions that contain zinc. Coating titanium with zinc using different surface modification procedures increased bone apposition around the surface modified material based on mechanical testing in rabbit models [189,190,191], histomorphometric measurement in a rat femur model [192], µCT and mechanical testing in a rat tibia model [193], and by dynamic and static histomorphometry, µCT, and mechanical testing in an osteoporotic rat femur model [194]. Bone formation was compared between uncoated titanium alloy rods and either calcium silicate coated or zinc-doped calcium silicate coated titanium alloy rods in a rabbit femur cortical defect model [195]. The bone-implant contact rate was approximately 70% for titanium alloy roads coated with zinc-doped calcium silicate and less than 30% for either control material indicating that the zinc increased bone formation and apposition. Similar results were obtained in an osteoporotic rabbit model using similarly coated titanium alloy rods [196]. A comparative study used the osteoporotic rat model to compare bone formation around titanium rods implanted in the femoral canal and coated with hydroxyapatite (HA) or HA containing 10% relative to calcium of zinc (ZnHA), magnesium (MgHA), or strontium (SrHA) [197]. Histomorphometry, µCT, and mechanical testing found that ZnHA promoted more bone formation than HA, but that MgHA and SrHA promoted more bone formation than ZnHA.

Bone formation is also enhanced around zinc-substituted calcium ceramics. Chou et al. found that bone formation in rat tibia defects filled with zinc-substituted ß-tricalcium phosphate (ZnTCP) was greater than those filled with unsubstituted ß-tricalcium phosphate (TCP) [198]. Yu et al. compared mesoporous hydroxyapatite (HA) and zinc-substituted HA (ZnHA) microspheres embedded in a collagen matrix as bone graft materials [199]. The ZnHA particles released most of the zinc after 5 days in vitro. In vivo, the graft material containing the ZnHA produced approximately 1/3 more bone in a rat femoral metaphysis defect model than HA graft material. Kawamura et al. used a rabbit femur defect model to illustrate that bone formation around ZnTCP/HA cylinders was zinc dose dependent and that the optimal zinc dose was 0.3 weight percent zinc [200,201]. Similarly, Luo et al. tested the osteogenic ability of sintered TCP blocks made with different amounts of zinc chloride (0, 5, 15, and 45 mmol ZnCl_2_ per 100 g TCP) [202]. The blocks were inserted into canine paraspinal muscles and osteogenesis was measured by histomorphometry. No bone formation was detected in the implantation sites for the lowest zinc doses, but 20% and 50% of the implantation site areas were bone in the sites treated with 15 and 45 mmol ZnCl_2_/100 g TCP granules, respectively. The osteoconductivity of TCP, ZnTCP, and zinc and strontium substituted ß-TCP (ZnSrTCP) cement was compared in a pig tarsal cortical defect model [109]. After 2 months, the ZnTCP cement performed better than the TCP cement, while the ZnSrTCP cement performed the best with 50% of the defect area filled with new bone. A meta-analysis of 18 animal studies examining the effects of zinc-incorporated calcium phosphate on bone repair concluded that the zinc-calcium phosphate did not affect biocompatibility, delayed graft resorption, and promoted osteoconduction [203]. Finally, in a clinical study, Bhardwaj et al. used ZnHA granules to treat periodontal bone defects and found that the ZnHA granules performed significantly better than HA granules by reducing defect volume while improving tooth clinical attachment values and radiographic evidence of bone formation [204]. In contrast and despite the long-term biocompatibility of zinc-substituted HA [205], ceramic cylinders of mixed ZnHA/TCP were no more osteogenic than cylinders of unsubstituted HA/TCP when implanted in rabbit tibia [206]. Fernandes et al. also found similar amounts of bone formation in critically sized rat calvarial defects between ZnHA and untreated groups [207]. 

Zinc and zinc-based alloys have substantial potential for use as biodegradable implants. Zinc metal is biodegradable but lacks sufficient mechanical properties to be useful for load-bearing applications [208,209]. Consequently, Guo et al. tested whether pure zinc membranes (30 µm thick) could be used for guided bone regeneration procedures in a rat calvaria defect model instead of titanium membrane [210]. After 28 days in Hank’s solution, zinc membrane had an approximate 33% weight loss, while titanium membrane had less than 5% weight loss. In vivo, more bone formation was measured in the defects covered with zinc membrane than uncovered and the amount of bone formed was comparable to titanium. In contrast to pure zinc, zinc alloys containing copper, magnesium, calcium, titanium, or combinations of these metals have improved mechanical properties while still maintaining acceptable degradation profiles and low cytotoxicity to osteoblast cell models [211,212]. Li et al. produced zinc alloys containing 1% magnesium (Zn-1Mg), calcium (Zn-1Ca), or strontium (Zn-1Sr) and measured changes in mechanical behavior as well as biological responses to each alloy [213]. The alloys all produced dramatic improvements in mechanical properties with solid in vitro degradation rates and low cytotoxicity. In vivo, bone formation occurred around the alloys implanted into mouse femora, but no control implant was used as a comparator. In contrast, Pina et al. found in a canine study that zinc alloy composed of 80% Zn, 18% Al, and 2% Cu (zinalco) negatively affected healing of femur osteotomies [214]. While 5 dogs treated with an intramedullary 316 stainless steel rod were able to weight bear on the affect hindlimb, none of the 10 dogs treated with the zinalco rod were able to weight bear on the affected hindlimb. Radiographic and scanning electron microscopy of the specimens noted disorganized callus formation and altered bone microstructure in the zinalco treated specimens. Whether the apparent negative effects on healing were caused by excessive zinc or by the aluminum and copper is unknown. 

Locally delivered zinc as a bolus or in a carrier matrix to a site in need of bone formation has only been recently investigated (Figure 3). Wey et al. injected a zinc chloride solution into the femoral canal of rats to deliver 0, 1, or 3 mg/kg zinc chloride just prior to fracture [215]. After 4 weeks of healing, torsional mechanical testing showed that the fracture calluses treated with 3 mg/kg of zinc chloride had significantly better structural (torque and rigidity) and material (shear stress and modulus) than the saline control or 1 mg/kg zinc chloride dose. Peak torque was 68% greater in the 3 mg/kg zinc chloride treated fracture calluses as compared to the saline controls. At 7 days after fracture, callus cartilage, cell proliferation, and IGF-1 and VEGF levels were significantly greater in the 3 mg/kg zinc chloride treated fractures than in saline controls. Krell et al. also delivered either a 1 or 3 mg/kg dose of zinc chloride directly to rat femur fractures but the zinc chloride solution was mixed with calcium sulfate and the paste was injected into the femoral canal just prior to fracture [216]. The calcium sulfate was used to slow the release of zinc. Controls included fractured femurs injected with saline or calcium sulfate paste containing no added zinc. At 4 weeks after fracture, the rat femurs treated with 1 mg/kg of zinc chloride showed dramatic improvements in fracture callus structural (torque and rigidity) and material (shear stress and shear modulus) properties, with a 60% increase in peak torque compared to the calcium sulfate control. Histology showed that fracture calluses in the 1 mg/kg zinc chloride treatment group had almost twice as much cartilage at 2 weeks after fracture than the calcium sulfate controls. In contrast, no significant effects of the 3 mg/kg zinc chloride in calcium sulfate dose on fracture callus mechanical properties was detected. Using a rat posterolateral spinal fusion (PLF) model, Koerner et al. also found that delivering zinc chloride in a calcium sulfate carrier was effective for promoting spinal fusion [217]. Fusion sites were treated with calcium sulfate pellets to deliver 0.5 or 1 mg/kg of zinc chloride. Both doses significantly improved fusion based on manual palpation, radiographic scoring and µCT quantitation of fusion bed bone volume. Together, these data indicate that zinc positively affects healing within a narrow dose range that can be manipulated through use of an excipient or carrier.

## 8. Future Directions

Three main avenues of research support development of zinc as a therapeutic agent for promoting bone regeneration. First are the clinical and experimental studies showing that dietary zinc insufficiency inhibits skeletal growth and alters bone quality. Normally, bone regeneration required to heal a fracture occurs by endochondral ossification during which a cartilage anlage is formed and replaced with bone. Endochondral ossification is also the primary developmental mechanism involved in forming the appendicular skeleton and in normal post-natal bone growth. Hence, if dietary zinc is critical for skeletal growth and bone quality, then zinc could also affect bone regeneration and more specifically, endochondral ossification in adults. Second, experiments examining effects of zinc on osteoblasts and chondrocytes show that zinc promotes the proliferation and activity of these cells, which are essential for endochondral ossification. Zinc also impairs osteoclast formation and activity. While osteoclast activity is important for successful endochondral ossification and remodeling of newly made bone, reduced osteoclast activity and increased chondrocyte and osteoblast activity would favor the early stages of bone regeneration. Finally, several experiments directly tested and found that zinc can promote bone regeneration via endochondral ossification or by stimulating direct bone formation (intramembranous ossification).

Future research directions will likely involve defining the cellular and molecular mechanisms that zinc affects to promote bone regeneration. As noted above, zinc has been shown to affect the activity of the major skeletal cell populations. Zinc also affects other cellular and physiological processes that would likely impact bone regeneration. Specifically, zinc has multiple effects on insulin production and signaling [33,34]. Zinc also can modulate the immune system which is increasingly recognized as a significant regulator of bone homeostasis and regeneration [104,218,219,220]. Use of discovery-based omics approaches to identifying pathways regulating bone regeneration and that are modulated by zinc is an attractive approach. However, analysis of ZIP and ZnT mutations in mice are already providing novel insights into the normal role of zinc in skeletal development and bone homeostasis [30,139,151,221]. Use of ZIP and ZnT genetically modified mice is likely to provide tools that can dissect the molecular pathways used by zinc during bone regeneration. 

Another future research direction will be defining optimal therapeutic approaches for using zinc to promote bone regeneration. Bone regeneration occurs naturally following fracture and methods that hasten or improve regeneration would likely reduce complications associated with temporary disability, delayed healing, and non-union. Bone regeneration is also vital for treating other clinical conditions including spinal and joint arthrodesis and healing of large bone defects caused from disease or trauma. Methods that can deliver the proper zinc dose over an optimal time span to effect successful healing are needed. Development of zinc-based therapeutics could be guided by a better understanding of the cellular and molecular mechanisms through which zinc acts. In the absence of such knowledge, zinc-based therapeutics will need to be developed based on the requirements of the clinical application and by experimentation to determine optimal dose regimens. In the case of a simple bone fracture, a zinc therapeutic may come in the form of an injectable or locally applied matrix in which the dose of zinc is controlled by the amount of zinc-matrix used. The duration of dosing would rely upon the solubility of the zinc salt used, zinc diffusion from the matrix, or by how often the zinc-matrix is applied as in the case of an injectable therapeutic. However, an injectable or locally applied zinc-matrix therapeutic would be unlikely to provide sufficient mechanical stabilization of the fracture, which instead would be provided by casting or application of fixators to the fractured bone. When treating large bone defects or inducing arthrodesis between bones, use of an osteoconductive material with significant mechanical properties would likely provide a better starting material for producing a zinc therapeutic. In this latter case, zinc dose would be controlled by the amount of zinc absorbed by the material or the amount of zinc substituted into the material during synthesis such as in the case of TCP and HA. The duration of zinc dosing would rely upon zinc release from the material, dissolution of the material, or both. An additional consideration for either approach is that the zinc released from the zinc-matrix or zinc-material will preferentially bind to nearby bone and create a secondary zinc source that can likely affect bone regeneration [215]. In other instances, use of fixation devices made of zinc alloys or that are coated in some fashion with zinc would likely promote appositional bone growth around the fixation device. All these potential zinc therapeutic approaches have shown some level of success. Further optimization and testing in large animal models are needed to spur commercial interest and move zinc therapeutics into clinical use.
